# Staphylococcus aureus Alpha-Toxin Is Conserved among Diverse Hospital Respiratory Isolates Collected from a Global Surveillance Study and Is Neutralized by Monoclonal Antibody MEDI4893

**DOI:** 10.1128/AAC.00357-16

**Published:** 2016-08-22

**Authors:** David E. Tabor, Li Yu, Hoyin Mok, Christine Tkaczyk, Bret R. Sellman, Yuling Wu, Vaheh Oganesyan, Tim Slidel, Hasan Jafri, Michael McCarthy, Patricia Bradford, Mark T. Esser

**Affiliations:** a Applied Immunology and Microbiology, MedImmune LLC, Mountain View, California, USA; b Statistical Sciences, MedImmune LLC, Gaithersburg, Maryland, USA; c Infectious Disease-Vaccine Research, MedImmune LLC, Gaithersburg, Maryland, USA; d Clinical Pharmacology and DMPK, MedImmune LLC, Gaithersburg, Maryland, USA; e Antibody Discovery and Protein Engineering, MedImmune LLC, Gaithersburg, Maryland, USA; f Research Bioinformatics, MedImmune LLC, Cambridge, United Kingdom; g Infectious Disease Clinical Research, MedImmune LLC, Gaithersburg, Maryland, USA; h Infectious Disease Innovative Medicines, MedImmune LLC, Gaithersburg, Maryland, USA; i Global Medicines Development, AstraZeneca, Waltham, Massachusetts, USA

## Abstract

Staphylococcus aureus infections lead to an array of illnesses ranging from mild skin infections to serious diseases, such endocarditis, osteomyelitis, and pneumonia. Alpha-toxin (Hla) is a pore-forming toxin, encoded by the *hla* gene, that is thought to play a key role in S. aureus pathogenesis. A monoclonal antibody targeting Hla, MEDI4893, is in clinical development for the prevention of S. aureus ventilator-associated pneumonia (VAP). The presence of the *hla* gene and Hla protein in 994 respiratory isolates collected from patients in 34 countries in Asia, Europe, the United States, Latin America, the Middle East, Africa, and Australia was determined. Hla levels were correlated with the geographic location, age of the subject, and length of stay in the hospital. *hla* gene sequence analysis was performed, and mutations were mapped to the Hla crystal structure. S. aureus supernatants containing Hla variants were tested for susceptibility or resistance to MEDI4893. The *hla* gene was present and Hla was expressed in 99.0% and 83.2% of the isolates, respectively, regardless of geographic region, hospital locale, or age of the subject. More methicillin-susceptible than methicillin-resistant isolates expressed Hla (86.9% versus 78.8%; *P* = 0.0007), and S. aureus isolates from pediatric patients expressed the largest amounts of Hla. Fifty-seven different Hla subtypes were identified, and 91% of the isolates encoded an Hla subtype that was neutralized by MED4893. This study demonstrates that Hla is conserved in diverse S. aureus isolates from around the world and is an attractive target for prophylactic monoclonal antibody (MAb) or vaccine development.

## INTRODUCTION

Staphylococcus aureus causes serious infections that increase morbidity and mortality. Especially life-threatening conditions are hospital-associated pneumonia (HAP) and ventilator-associated pneumonia (VAP), caused by S. aureus (1 – 4). Globally, approximately 10 million patients are admitted annually to intensive care units (ICUs) in major health care centers, and according to the Centers for Disease Control and Prevention, S. aureus accounts for more than 40% of VAP cases in the United States (5). ICU length of stay is extended an average of 17 days after the onset of S. aureus pneumonia, and attributable mortality can reach 30% despite the use of antibiotics (6).

S. aureus secretes a number of virulence factors to evade the host immune response and contribute to pathogenesis. They include superantigens, leukocidins, complement evasion proteins, and the cytolytic toxin Hla (7 – 9). Hla is a 33-kDa pore-forming toxin encoded by the *hla* gene (10) that forms heptameric pores in host cell membranes, leading to lysis of the cell (11). Even at sublytic levels, Hla has been shown to affect innate immune effector cells, stimulate a hyperinflammatory response characteristic of bacterial pneumonia, and disrupt epithelial and endothelial barriers (12, 13). Hla expression is controlled by a complex regulatory network (14 – 16), and its expression has been reported to be upregulated during infection (17). Studies using isogenic *hla* knockout mutants have shown Hla to be a key virulence factor in animal models of sepsis, skin and soft tissue infections, and pneumonia (11, 13, 18). Furthermore, active and passive immunization approaches have been effective in preventing skin and soft tissue infections, pneumonia, and death in animal models of S. aureus disease (19 – 21), and epidemiological studies have reported that high levels of anti-Hla serum antibodies correlate with protection from infection or severe disease (22 – 24). Consequently, Hla is being evaluated as a target for vaccination and passive immunotherapies against diseases caused by S. aureus (19, 25, 26).

MEDI4893 is a human monoclonal antibody (MAb) with Hla-neutralizing activity currently in clinical development for the prevention of VAP (27). Hla neutralization by MEDI4893 has been reported to protect the lung epithelium and innate immune cells (e.g., alveolar macrophages) from Hla-mediated damage, thereby promoting bacterial clearance and dampening the hyperinflammatory response characteristic of bacterial pneumonia, leading to improved outcomes in preclinical acute-pneumonia models (25, 28, 29). To better understand the prevalence of Hla, we characterized the presence of the *hla* gene, Hla mutations, expression levels, and the relative susceptibility to MEDI4893 in methicillin-sensitive S. aureus (MSSA) and methicillin-resistant S. aureus (MRSA) isolates collected as part of an international surveillance program. The study was designed to analyze 500 MSSA and 500 MRSA respiratory isolates collected from hospitals in Asia, Europe, the United States, Latin America, the Middle East, Africa, and Australia.

## MATERIALS AND METHODS

### S. aureus isolates.

Isolates of S. aureus were analyzed as part of a collection from an international antibiotic resistance surveillance program. The S. aureus isolates were stored at −80°C until use. Basic demographic data (age, sex, hospital location, sample type, and length of stay) were provided for each isolate using a unique study number that was delinked from any patient identification.

### *hla* PCR, Sanger sequencing, whole-genome sequencing, and phylogenetic analysis.

S. aureus *hla* PCR and Sanger sequencing were performed as previously described (30). The forward and reverse PCR primers were F1, 5′-TGTCTCAACTGCATTATTCTAAATTG-3′, and R1, 5′-CATCATTTCTGATGTTATCGGCTA-3′. PCR amplicons were sequenced with the BigDye Terminator cycle-sequencing kit v3.1 (Applied Biosystems) using the F1, R1, F2 (5′-TGCAAATGTTTCAATTGGTCATAC-3′), F3 (5′-CAGATTCTGATATTAATATTAAAAC-3′), and R2 (5′-TCCCCAATTTTGATTCACCA-3′) primers (31). Libraries were generated using the Nextera XT DNA Library Preparation kit, and sequencing was performed on a MiSeq instrument (Illumina). Consensus sequences were generated by reference mapping, and *de novo* assembly was performed using CLC Genomics Workbench (CLC bio). The *hla* gene sequences obtained from 984 isolates were aligned using MUSCLE, and the phylogenetic tree was generated using the neighbor-joining method in MEGA5 (32, 33). Consensus DNA sequences were translated into protein sequences, and amino acid substitutions were identified by using USA 300 CP000255_SAUSA300_1058 as a reference sequence (34).

### Multilocus sequence typing.

Multilocus sequence types (MLSTs) were determined using CLCBio Genomic Workbench 8.0.1 and the MLST schema for S. aureus (http://www.pubmlst.org). Concatenated MLST alleles were used to construct phylogenetic trees using the neighbor-joining method in MEGA5 (32, 33).

### Hla quantification by enzyme-linked immunosorbent assay (ELISA).

Hla quantification was performed for S. aureus culture supernatants as previously described (30). Briefly, a single colony from a fresh tryptic soy agar (TSA) plate was inoculated into 3 ml of tryptic soy broth (TSB) (Becton Dickinson) and incubated overnight with shaking (220 rpm) at 37°C. An aliquot of the overnight culture was diluted in 10 ml of TSB (optical density at 600 nm [OD_600_], ∼0.1) in a 150-ml flask and incubated at 37°C at 220 rpm for 16 h. The bacteria were then pelleted by centrifugation, and the supernatant was removed, filter sterilized, and stored at −80°C. Ninety-six-well high-bind plates (VWR International) were coated overnight at 4°C with MEDI4893 (0.1 μg/ml) in 0.2 M carbonate-bicarbonate buffer. Following washing with phosphate-buffered saline (PBS)-0.1% Tween 20 (PBST), the plates were blocked with PBST plus 5% bovine serum albumin (BSA) for 1 h at room temperature (RT). Overnight S. aureus culture supernatants (1:1,000 dilution) and a purified Hla reference standard were added and incubated at RT for 1 h. The plates were washed 4 times with PBST and incubated for 1 h with an anti-Hla rabbit IgG (1 μg/ml), and the rabbit IgG was detected with a horseradish peroxidase (HRP)-conjugated goat anti-rabbit IgG (1:10,000; Jackson ImmunoResearch). Following a 1-h incubation at RT, the plates were washed 4 times with PBST, and antibody binding was detected with 100 μl of SureBlue TMB substrate (KPL), followed by neutralization with 100 μl of TMB Stop Solution (KPL). The OD_450_ was measured on a Sunrise plate reader (Tecan).

### Rabbit RBC Hla hemolytic assay and neutralization assay.

Hla hemolytic activity was measured using a rabbit red blood cell (RBC) lysis assay, as previously described (28). Supernatants obtained as detailed above were serially 2-fold diluted from 1:5 to 1:12,800 in 50 μl PBS in a U-bottom 96-well plate, followed by addition of 50 μl of 5% PBS-washed RBCs (PelFreeze). After a 90-min incubation at 37°C, the plate was centrifuged at 1,500 rpm for 2 min, 50 μl of supernatant was transferred to a 96-well flat-bottom plate, and the OD_450_ was determined. The Hla level (in hemolytic units [HU] per milliliter) was defined as the inverse of the dilution causing 50% hemolysis. Sterile distilled water served as the 100% hemolysis control (positive control), and 1× PBS was a negative control. Supernatants from isolates with hemolytic activity titers greater than that of the *hla* knockout strain were tested for neutralization by MEDI4893. A 2-fold serial dilution, starting at 1:5, of S. aureus supernatants was incubated with 25 μl of MEDI4893 (200 μg/ml) and added to the RBCs. Supernatants from selected isolates that were not completely neutralized by MEDI4893 were also incubated with anti-chicken ovalbumin (OVA)-purified rabbit IgG, anti-γ-hemolysin B (HlgB)-purified rabbit IgG, or anti-Hla-purified rabbit IgG at 200 μg/ml or in combination. After a 90-min incubation at 37°C, the plates were spun, the supernatants were collected, and the OD_450_ was determined. Complete neutralization was determined if hemolytic activity in the presence of MEDI4893 was below either the assay lower limit of detection or the hemolytic activity background as determined using an *hla* knockout strain.

### Protein structure visualization.

Amino acid substitutions were mapped to both the monomer and heptamer Hla structures using PyMOL v1.6 (http://www.pymol.org/). The frequencies of amino acid substitutions in relation to the USA300 reference strain were shown using the following color scale: cyan, 1 to 7; green, 13 to 17; ruby, 30 to 36; brown, 62; magenta, 133 to 140; and red, >450. The MEDI4893 binding region is shown in white.

### Statistical methods.

Stratified random sampling was performed to select the original 500 MSSA and 500 MRSA isolates, with 125 isolates each of MSSA and MRSA from each of four regions (Asia, Europe, the United States, and the rest of the world [ROW]). Random sampling was controlled over the variables of country, sample type, and hospital location. Fisher's exact test was used for comparison of the proportions of categorical variables, a Wilcoxon rank sum test was used for comparison of patient age and Hla secretion levels between different groups, and a two-sample Poisson rate test was used to compare the occurrence of MSSA versus MRSA and subtypes of MSSA versus MRSA. Pearson's chi-squared test was used to test whether MSSA and MRSA isolates were more likely to come from male than from female patients. Sampling and statistical analyses were performed using SAS 9.3 (SAS) and R (http://www.r-project.org). The figures were generated using GraphPad Prism 6 (GraphPad).

## RESULTS

### Patient demographics and S. aureus characterization.

**FIG 1:**
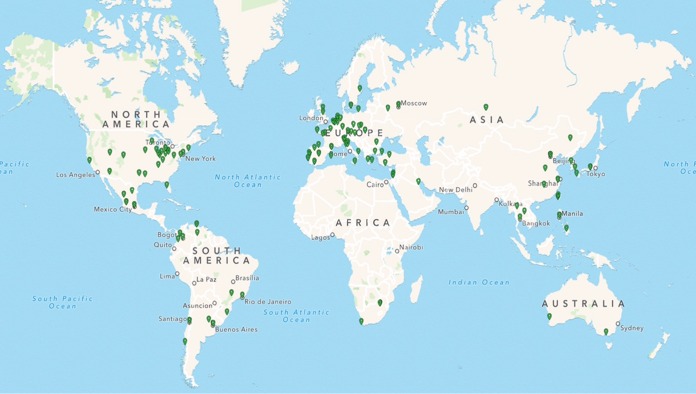
Hospital locations in the S. aureus global surveillance study. The map shows the hospital locations in 34 countries that provided S. aureus respiratory isolates for characterization.

To better understand the global prevalence of the *hla* gene and Hla expression in S. aureus respiratory isolates, we randomly selected 500 MSSA and 500 MRSA isolates from a total of 2,068 MSSA and 3,434 MRSA isolates collected during 2012 and 2013 as part of a prospective 5-year global antibacterial resistance surveillance program. One hundred and twenty-five identified MSSA and MRSA respiratory isolates from each of four geographic regions (Asia, Europe, the United States, and the rest of the world) representing 34 countries (Fig. 1) were selected for culture and characterization. Of the 1,000 isolates selected, 2 isolates originally classified as MSSA by culture were determined to be Staphylococcus epidermidis and 4 isolates did not grow, leaving 994 for analysis (Table 1). All the isolates were derived from endotracheal aspirates, bronchoalveolar brush samples, lavage samples, or sputum and came from nine different departments in the participating hospitals. Isolates were also categorized as coming from patients who had been in the hospital for less than or more than 48 h and male or female and by the age of the patient. The majority of the isolates came from either general medicine, ICU, general surgery, or surgical ICU, and less than 1% came from outpatient departments. Interestingly, the isolates from the pediatric departments were more likely to be MSSA than MRSA (46 versus 23; *P* = 0.0040) (Table 1).

**TABLE 1:** Patient demographics and summary of *hla* presence and alpha-toxin expression levels

Variable	No. (%) of samples										*P* value (MRSA vs MSSA)
	MRSA					MSSA					
	Asia (*n* = 125)	Europe (*n* = 125)	United States (*n* = 125)	Row (*n* = 125)	All (*n* = 500)	Asia (*n* = 123)	Europe (*n* = 125)	United States (*n* = 125)	Row (*n* = 125)	All (*n* = 498)	
Yr collected											
2012	118 (94.4)	80 (64.0)	77 (61.6)	82 (65.6)	357 (71.4)	119 (96.7)	68 (54.4)	74 (59.2)	75 (60.0)	336 (67.5)	0.1918
2013	7 (5.6)	45 (36.0)	48 (38.4)	43 (34.4)	143 (28.6)	4 (3.3)	57 (45.6)	51 (40.8)	50 (40.0)	162 (32.5)	
Facility name											
Emergency room	8 (6.4)	5 (4.0)	2 (1.6)	13 (10.4)	28 (5.6)	7 (5.7)	3 (2.4)	2 (1.6)	11 (8.8)	23 (4.6)	0.5657
General unspecified ICU	2 (1.6)	14 (11.2)	6 (4.8)	8 (6.4)	30 (6.0)	6 (4.9)	12 (9.6)	6 (4.8)	7 (5.6)	31 (6.2)	0.8957
Medicine, general	67 (53.6)	56 (44.8)	47 (37.6)	48 (38.4)	218 (43.6)	66 (53.7)	45 (36.0)	44 (35.2)	39 (31.2)	194 (39.0)	0.1397
Medicine, ICU	25 (20.0)	25 (20.0)	38 (30.4)	33 (26.4)	121 (24.2)	20 (16.3)	21 (16.8)	37 (29.6)	35 (28.0)	113 (22.7)	0.6012
Outpatient	0 (0.0)	0 (0.0)	0 (0.0)	0 (0.0)	0 (0.0)	0 (0.0)	0 (0.0)	1 (0.8)	0 (0.0)	1 (0.2)	0.4990
Pediatric, general	0 (0.0)	6 (4.8)	4 (3.2)	3 (2.4)	13 (2.6)	3 (2.4)	15 (12.0)	2 (1.6)	4 (3.2)	24 (4.8)	0.0673
Pediatric, ICU	3 (2.4)	2 (1.6)	2 (1.6)	3 (2.4)	10 (2.0)	3 (2.4)	6 (4.8)	6 (4.8)	7 (5.6)	22 (4.4)	0.0320
Surgery, general	12 (9.6)	8 (6.4)	6 (4.8)	6 (4.8)	32 (6.4)	12 (9.8)	8 (6.4)	8 (6.4)	6 (4.8)	34 (6.8)	0.8003
Surgery, ICU	8 (6.4)	9 (7.2)	20 (16.0)	11 (8.8)	48 (9.6)	6 (4.9)	15 (12.0)	19 (15.2)	16 (12.8)	56 (11.2)	0.4089
Body location											
Bronchial brushing	1 (0.8)	9 (7.2)	4 (3.2)	12 (9.6)	26 (5.2)	1 (0.8)	13 (10.4)	4 (3.2)	6 (4.8)	24 (4.8)	0.8848
Bronchoalveolar lavage	1 (0.8)	15 (12.0)	21 (16.8)	7 (5.6)	44 (8.8)	6 (4.9)	16 (12.8)	33 (26.4)	15 (12.0)	70 (14.1)	0.0096
Endotracheal aspirate	6 (4.8)	41 (32.8)	32 (25.6)	61 (48.8)	140 (28.0)	7 (5.7)	40 (32.0)	24 (19.2)	61 (48.8)	132 (26.5)	0.6191
Lungs	0 (0.0)	0 (0.0)	1 (0.8)	0 (0.0)	1 (0.2)	0 (0.0)	1 (0.8)	0 (0.0)	2 (1.6)	3 (0.6)	0.3732
Sputum	117 (93.6)	60 (48.0)	67 (53.6)	45 (36.0)	289 (57.8)	109 (88.6)	55 (44.0)	64 (51.2)	41 (32.8)	269 (54.0)	0.2511
Gender											
Female	33 (26.4)	34 (27.2)	45 (36.0)	47 (37.6)	159 (31.8)	50 (40.7)	60 (48.0)	46 (36.8)	48 (38.4)	204 (41.0)	0.0030
Male	82 (65.6)	90 (72.0)	76 (60.8)	76 (60.8)	324 (64.8)	67 (54.5)	65 (52.0)	76 (60.8)	76 (60.8)	284 (57.0)	0.0137
Not specified	10 (8.0)	1 (0.8)	4 (3.2)	2 (1.6)	17 (3.4)	6 (4.9)	0 (0.0)	3 (2.4)	1 (0.8)	10 (2.0)	0.2413
Hospital stay											
≥48 h	96 (76.8)	66 (52.8)	74 (59.2)	56 (44.8)	292 (58.4)	82 (66.7)	68 (54.4)	55 (44.0)	42 (33.6)	247 (49.6)	0.0063
<48 h	22 (17.6)	31 (24.8)	26 (20.8)	33 (26.4)	112 (22.4)	37 (30.1)	31 (24.8)	35 (28.0)	45 (36.0)	148 (29.7)	0.0094
Not specified	7 (5.6)	28 (22.4)	25 (20.0)	36 (28.8)	96 (19.2)	4 (3.3)	26 (20.8)	35 (28.0)	38 (30.4)	103 (20.7)	0.5797
Age (yr)											
Median (min, max)	72 (0, 98)	64 (0, 93)	61 (0, 94)	57 (0, 96)	63 (0, 98)	63 (0, 90)	50 (0, 94)	58 (0, 90)	49 (0, 89)	55 (0, 94)	<0.0001
0–20	4 (3.2)	11 (8.8)	11 (8.8)	17 (13.6)	43 (8.6)	12 (9.8)	26 (20.8)	12 (9.6)	25 (20.0)	75 (15.1)	0.0017
21–40	7 (5.6)	14 (11.2)	12 (9.6)	18 (14.4)	51 (10.2)	15 (12.2)	27 (21.6)	16 (12.8)	27 (21.6)	85 (17.1)	0.0017
41–60	26 (20.8)	31 (24.8)	39 (31.2)	33 (26.4)	129 (25.8)	30 (24.4)	24 (19.2)	42 (33.6)	32 (25.6)	128 (25.7)	>0.9999
61–80	60 (48.0)	50 (40.0)	46 (36.8)	36 (28.8)	192 (38.4)	41 (33.3)	42 (33.6)	38 (30.4)	31 (24.8)	152 (30.5)	0.0094
81–100	28 (22.4)	19 (15.2)	17 (13.6)	21 (16.8)	85 (17.0)	25 (20.3)	6 (4.8)	17 (13.6)	10 (8.0)	58 (11.6)	0.0186
*hla* gene											
Negative	6 (4.8)	2 (1.6)	1 (0.8)	0 (0.0)	9 (1.8)	1 (0.8)	0 (0.0)	0 (0.0)	0 (0.0)	1 (0.2)	0.0209
Positive	119 (95.2)	122 (97.6)	123 (98.4)	124 (99.2)	488 (97.6)	122 (99.2)	125 (100.0)	125 (100.0)	124 (99.2)	496 (99.6)	0.0123
Did not grow	0 (0.0)	1 (0.8)	1 (0.8)	1 (0.8)	3 (0.6)	0 (0.0)	0 (0.0)	0 (0.0)	1 (0.8)	1 (0.2)	0.6242
Hla ELISA level											
Negative	44 (35.2)	26 (20.8)	13 (10.4)	20 (16.0)	103 (20.6)	21 (17.1)	13 (10.4)	14 (11.2)	16 (12.8)	64 (12.9)	0.0012
Positive	81 (64.8)	98 (78.4)	111 (88.8)	104 (83.2)	394 (78.8)	102 (82.9)	112 (89.6)	111 (88.8)	108 (86.4)	433 (86.9)	0.0007
Positive, <1 μg/ml	44 (35.2)	46 (36.8)	38 (30.4)	39 (31.2)	167 (33.4)	31 (25.2)	29 (23.2)	33 (26.4)	24 (19.2)	117 (23.5)	0.0006
Positive, 1–10 μg/ml	36 (28.8)	42 (33.6)	64 (51.2)	50 (40.0)	192 (38.4)	50 (40.7)	63 (50.4)	61 (48.8)	46 (36.8)	220 (44.2)	0.0718
Positive, >10 μg/ml	1 (0.8)	10 (8.0)	9 (7.2)	15 (12.0)	35 (7.0)	21 (17.1)	20 (16.0)	17 (13.6)	38 (30.4)	96 (19.3)	<0.0001
Did not grow	0 (0.0)	1 (0.8)	1 (0.8)	1 (0.8)	3 (0.6)	0 (0.0)	0 (0.0)	0 (0.0)	1 (0.8)	1 (0.2)	0.6242

There were regional differences in the types of samples collected. Most notable was the fact that approximately 90% of the Asian isolates came from sputum samples. There were no obvious differences in the sample types with respect to frequencies of MSSA versus MRSA isolates, except that 14.1% of bronchoalveolar lavage (BAL) samples were MSSA versus 8.8% MRSA (*P* = 0.010). The majority of both the MSSA (64.8% versus 31.8%; *P* < 0.0001) and MRSA (57.0% versus 41.0%; *P* = 0.0002) isolates were from males versus females. More MRSA than MSSA isolates were from patients who were in the hospital for more than 48 h (58.4% versus 49.6%; *P* = 0.006). The age of the patients ranged from <12 months to 98 years, with the median age of MRSA patients being considerably higher than that of MSSA patients (63 years versus 55 years; *P* < 0.0001).

### The alpha-toxin gene *hla* and protein expression are conserved in diverse S. aureus isolates.

The *hla* gene was present in the majority of the MSSA (99.6%) and MRSA (97.6%) isolates (Table 1), demonstrating that the presence of the gene is highly conserved. Ten isolates were determined to be *hla* gene negative by both *hla*-specific PCR and whole-genome sequencing. Interestingly, 7 of the 10 *hla*-negative isolates were from a single hospital in Thailand. The majority of the isolates also expressed measurable Hla *in vitro*, but the level was higher for MSSA than for MRSA isolates (86.9% versus 78.8%; *P* = 0.0007). Furthermore, the MSSA strains produced higher levels of Hla than the MRSA strains (4.20 μg/ml versus 2.26 μg/ml; *P* < 0.0001), and a higher proportion of MSSA strains than of MRSA strains (19.3% versus 7.0%; *P* < 0.0001) expressed the largest amounts of Hla (>10 μg/ml) (Table 1).

**FIG 2:**
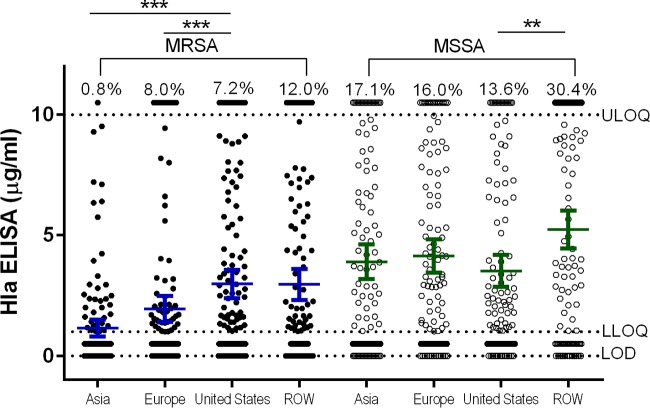
Alpha-toxin expression levels in MSSA and MRSA isolates by geographic region. Mean Hla ELISA levels of duplicate samples are shown for MSSA and MRSA isolates from the four geographic regions. The mean and 95% confidence interval (CI) are shown for each group. The percentages above the upper limit of quantitation (ULOQ) (10 μg/ml) represent the percentages of isolates within a group with a value of >10 μg/ml. The assay lower limit of quantitation (LLOQ) and limit of detection (LOD) are indicated by horizontal dotted lines. Statistically significant differences are indicated by asterisks: ***, *P* < 0.001; **, *P* < 0.01.

**FIG 3:**
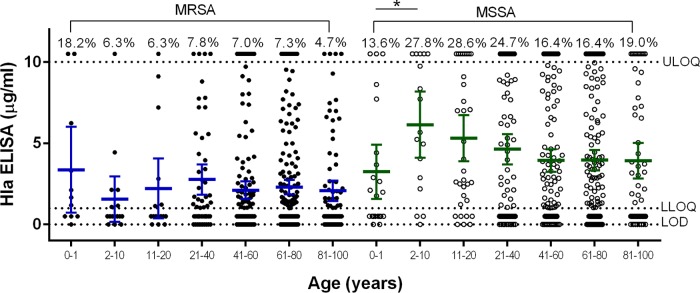
Alpha-toxin expression levels in MSSA and MRSA isolates by patient age. Mean Hla ELISA levels of duplicate samples are shown for MSSA and MRSA isolates from subjects of different ages. The mean and 95% CI are shown for each age group. The percentages above the upper limit of quantitation (10 μg/ml) represent the percentages of isolates within a group with a value of >10 μg/ml. The assay lower limit of quantitation and limit of detection are indicated by horizontal dotted lines. Statistically significant differences are marked by an asterisk: *, *P* < 0.05.

**FIG 4:**
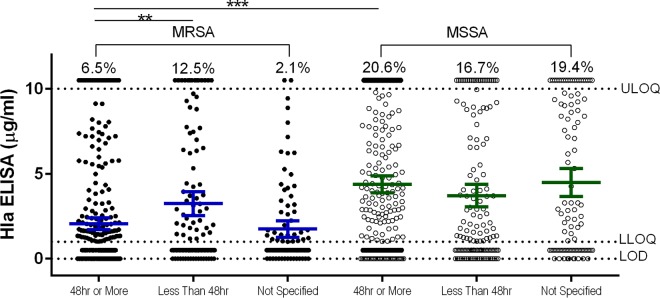
Alpha-toxin expression levels in MSSA and MRSA isolates by length of hospital stay. Mean Hla ELISA levels of duplicate samples are shown for MSSA and MRSA isolates from subjects with hospital lengths of stay less than or greater than 24 h. The mean and 95% CI are shown for each group. The percentages above the upper limit of quantitation (10 μg/ml) represent the percentages of isolates within a group with a value of >10 μg/ml. The assay lower limit of quantitation and limit of detection are indicated by horizontal dotted lines. Statistically significant differences are marked by asterisks: ***, *P* <0.001; **, *P* < 0.01.

Comparing Hla expression levels by geographic region revealed that a higher proportion of Asian, European, and ROW MSSA isolates than MRSA isolates from the same regions expressed detectable Hla, while U.S. isolates were more comparable in terms of relative Hla expression levels (Fig. 2). These data suggest that there are regional differences in Hla expression in MSSA and MRSA isolates from around the world. Because MSSA patients were on average younger than MRSA patients and MSSA isolates expressed larger amounts of Hla, the correlation between Hla expression levels and patient age was evaluated (Fig. 3). MSSA isolated from infants, adolescents, and young adults aged 0 to 20 years expressed the highest levels of Hla, suggesting these isolates were qualitatively different from the others. The relationship between Hla expression levels and length of stay in the hospital associated with MSSA and MRSA isolates was also determined (Fig. 4). For MRSA, isolates from patients with longer hospital stays were more likely to be Hla negative, and conversely, shorter stays were associated with isolates being Hla positive (*P* = 0.0030). These data suggest that there is a complex interplay between the infecting strain, antibiotic resistance, alpha-toxin, and the host immune response.

### Alpha-toxin subtypes.

**FIG 5:**
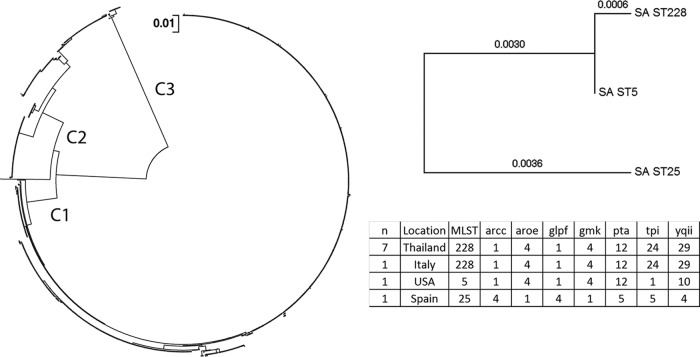
Phylogenetic tree of alpha-toxin subtypes. The evolutionary distances of the different *hla* types were computed using the maximum composite likelihood method and are shown as the number of base substitutions per site. The analysis was performed on 984 full-length nucleotide sequences, as sequences that contained gaps or missing sequence were not included. There were a total of 941 positions in the final data set. Evolutionary analyses were conducted in MEGA5. The 3 major clades (C1, C2, and C3) are indicated at their respective branch points. Multilocus sequence typing analysis was performed using whole-genome sequence data from 10 *hla* gene-negative isolates, MLST assignment and allele usage are listed in the table, and the seven genes in the MLST schema are shown in the table headers. Concatenated MLST alleles from the 10 *hla* gene-negative isolates were used to construct a phylogenetic tree using the neighbor-joining method in MEGA5, and branch length values are indicated. SA ST, S. aureus sequence type.

Sequence analysis revealed 82 different amino acid substitutions compared to the USA300 reference strain, which corresponded to 57 distinct Hla subtypes (Table 2) that grouped into three distinct clades (Fig. 5). Forty-seven MSSA and 27 MRSA subtypes were identified (*P* < 0.0133), with 17 subtypes in common between MSSA and MRSA strains (Table 2). In addition to amino acid changes, 50 isolates had a stop codon and 13 had a frameshift mutation in the *hla* gene. The majority (42 of 47) of the isolates with the previously described Q113 stop codon (34, 35) were MSSA strains (Table 2). The 6 most abundant MRSA subtypes comprised 90% of the MRSA isolates, and the 6 most abundant MSSA subtypes comprised 79% of the MSSA isolates. For MRSA, 18 of 29 (62%) subtypes had less than 3 members, and for MSSA, 33 of 47 (70%) subtypes had less than 3 members. These data show that not only the presence of Hla, but also the protein sequence is highly conserved.

**TABLE 2:**
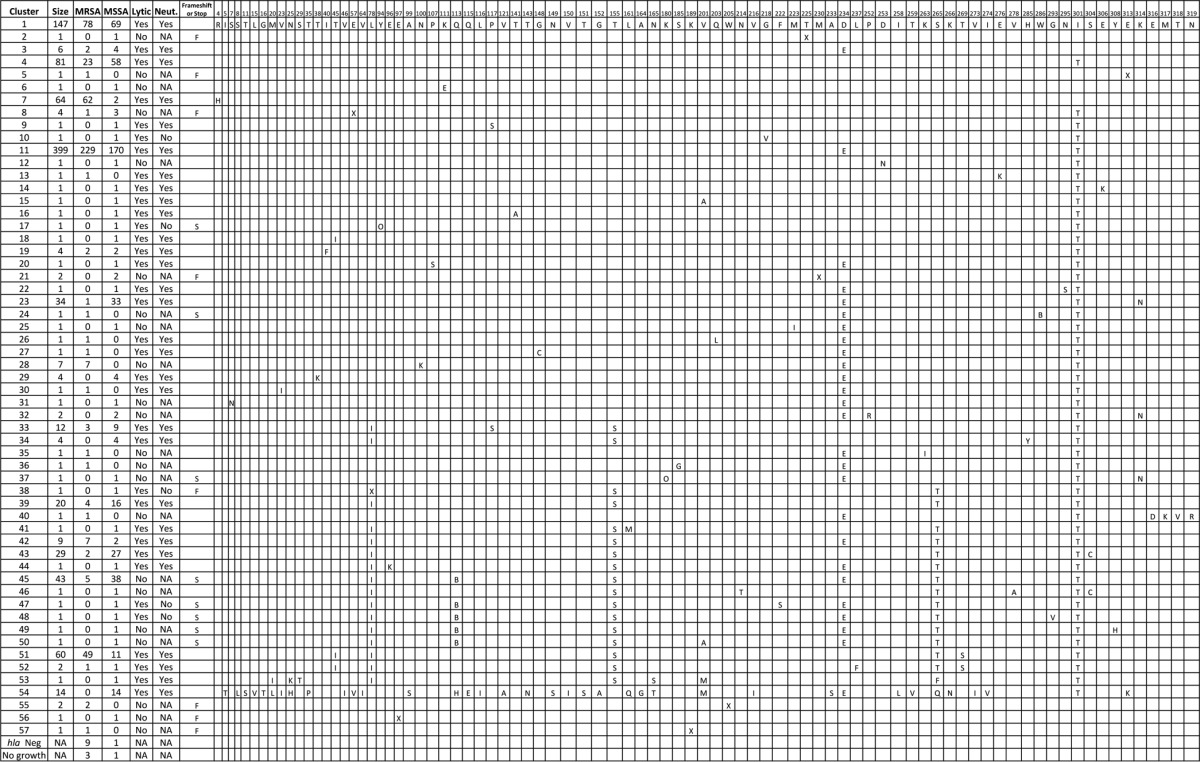
Alpha-toxin *hla* gene subtypes, amino acid variants, lytic activity, and MEDI4893 susceptibility

### Alpha-toxin monomer and heptamer.

**FIG 6:**
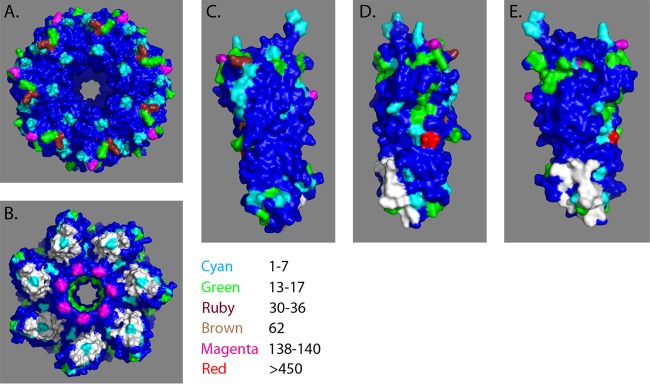
Alpha-toxin monomer and heptamer structures. (A) Hla heptamer, top view. (B) Hla heptamer, bottom view. (C) Hla monomer, 0°. (D) Hla monomer, 135°. (E) Hla monomer, 270°. The frequencies of amino acid substitutions in relation to the USA300 reference strain are shown using a color scale. The MEDI4893 binding region is shown in white.

The structures of the Hla monomer (35), the Hla heptamer (36), and the MEDI4893 binding region (35) have been previously described. The MEDI4893 binding region encompasses amino acid residues 203 to 226 and 287 to 297 (35) and neutralizes Hla by preventing Hla binding to cells (35). The 82 amino acid substitutions were color coded based on frequency and mapped to both the monomer and the heptamer structures (Fig. 6A to E) in relation to the MEDI4893 epitope (shown in white). This analysis showed that 6 out of the 82 amino acid mutations identified were within the MEDI4893 binding region, representing 19 different isolates. However, four out of these six amino acids do not directly participate in the interaction with the antibody (amino acids [aa] 203, 218, 223, and 295, representing 4 different isolates), and these mutations are not expected to be of consequence. The other two amino acid mutations either make very little contribution to the interaction or the amino acid substitution is conservative. The change of an N to a T at position 214 results in the loss of a weak hydrogen bond and represents 1 isolate. The V-to-I change at position 216, representing 14 isolates, is conservative and is also unlikely to affect MEDI4893 binding to Hla. In summary, the MEDI4893 binding region is highly conserved.

### Neutralization of Hla subtypes by MEDI4893.

Lastly, to determine whether the different Hla subtypes were susceptible or resistant to MEDI4893 neutralization, we tested whether MEDI4893 could neutralize culture supernatants from a panel of the 35 subtypes that had hemolytic activity (Table 2). These 35 hemolytic subtypes represent 908 of the 994 (91.4%) isolates. MEDI4893 neutralized the hemolytic activity in the culture supernatants of 30 of the 35 subtypes. The remaining 5 isolates, which showed lytic activity but were not neutralized by MEDI4893, belonged to subtypes 10, 17, 38, 47, and 48. In addition to Hla, rabbit RBCs are sensitive to lysis by γ-hemolysin (8). Using an anti-γ-hemolysin subunit B (HlgB)-purified rabbit IgG, hemolysis of 4 of these 5 isolates was shown to be due to γ-hemolysin (see Fig. S1a in the supplemental material). The observation that RBC lysis by subtype 38 supernatant was not inhibited by anti-HlgB IgG, anti-Hla IgG, MEDI4893, or a combination of the antibodies indicates that hemolysis by this isolate is not Hla or HlgB dependent but is likely due to another toxin(s), such as phenol-soluble modulins (PSMs) or δ-toxin (37, 38). Moreover, the hemolytic activities of 3 subtypes (39, 52, and 54) were partially neutralized by MEDI4893, but hemolysis was neutralized by more than 75% by a combination of MEDI4893 and anti-γ-hemolysin IgG, suggesting that both Hla and γ-hemolysin contribute to the hemolytic activity of these 3 clusters (see Fig. S1b in the supplemental material).

These data suggest that the MEDI4893 epitope is highly conserved and that MEDI4893 neutralizes the majority of Hla subtypes.

## DISCUSSION

This study analyzed 994 hospital S. aureus respiratory isolates from 34 countries collected from 2012 to 2013. Whereas others have characterized the presence of *hla* and Hla in S. aureus isolates (30, 39, 40), to our knowledge, this is the first prospective, global study to characterize Hla in relation to patient demographics and susceptibility to an Hla-neutralizing MAb. This work has some limitations. First, the type and duration of antibiotic treatment associated with these specimens are unknown, and second, patient outcomes were not collected and we therefore could not correlate clinical outcomes with a given Hla subtype or Hla level. Another practical limitation is that Hla expression was assessed *in vitro* using an ELISA that was optimized to ensure specificity at the expense of sensitivity and that might miss some low-expressing isolates. Additionally, these *in vitro* values may not represent the levels expressed during infection. Despite these limitations, this study provides valuable insights into the conservation of *hla* presence and Hla amino acid sequence and function in both MSSA and MRSA isolates from diverse geographic regions.

Independent of geography, patient age, or length of hospital stay, more MSSA isolates than MRSA isolates expressed Hla, as well as higher levels of Hla. This suggests that the genetic background of MSSA allows higher Hla expression and is consistent with a prior study in which Stulik et al. reported that MSSA respiratory isolates produced more Hla than MRSA isolates (41). The Hla subtype or specific amino acid substitutions did not correlate with Hla expression levels, as there were isolates with the same amino acid sequence that expressed low or high levels or no toxin. This observation is consistent with other reports showing that Hla production is controlled at the transcriptional level by a complex regulatory network (42, 43). Interestingly, the strains expressing the highest Hla levels were predominantly MSSA and came from the pediatric wards (Fig. 3). This finding is consistent with an increased incidence of MSSA in the pediatric population, suggesting that they may have been infected with community-acquired S. aureus as opposed to a hospital-acquired MRSA strain.

Phylogenetic analysis revealed that the 57 identified subtypes grouped into 3 major clades (Fig. 5). The two most populous clades are consistent with those described by Tavares et al. (40). Such similarity was anticipated given the overlap of sampling time frames and geographic locations. Our observation of a third, minor clade was likely due to the increased depth of sampling in our study. More Hla variation was discovered in MSSA than in MRSA isolates (43 versus 24 subtypes; *P* = 0.0136), which was presumably due to the relatively recent evolutionary emergence of methicillin resistance compared to the evolution of alpha-toxin. In addition to amino acid substitutions, 50 isolates had a stop codon. The Q113 stop codon has been described previously (40, 44) and has been associated with attenuated virulence in animal models and niche adaptation in humans (45). There were only 10 *hla* gene-negative isolates identified in this study. Interestingly, 7 of these 10 isolates were isolated from a single hospital in Thailand in 2012, whereas the other 3 were collected in 2013 from Spain, Italy, and the United States. Multilocus sequence typing analysis revealed that the seven isolates from Thailand (sequence type 228 [ST228]), the one from Italy (ST228), and the one from the United States (ST5) belonged to similar MLSTs and could be related (Fig. 5). These observations highlight the importance of continued surveillance during antibody or vaccine development.

The MEDI4893 binding region is comprised of amino acids 203 to 226 and 287 to 297, including the leader sequence, and the specific Hla contact residues are amino acids 209, 212, 213, 214, 215, 217, 226, 289, and 292 (32). The data presented here suggest the region is important for Hla activity, as only 19 isolates encoding full-length Hla with mutations in this region were identified (Table 2 and Fig. 6A to E). Only the single subtype 10 isolate that had lytic activity and was not neutralized by MEDI4893 had a mutation in the binding region (G218V). However, the supernatant from this isolate was also not neutralized by a polyclonal anti-Hla IgG, suggesting that the lytic activity was due to a hemolysin other than Hla. Previous work has shown that the MEDI4893 epitope is important for binding to cells (35), and the data presented here demonstrate that it is highly conserved.

In summary, we have shown that the presence of the Hla gene (*hla*) and the MEDI4893 epitope are highly conserved in respiratory isolates of S. aureus collected from hospitals as part of a global surveillance study. MSSA isolates were more likely to be Hla positive and to secrete larger amounts of Hla than MRSA isolates, and MEDI4893 neutralized the vast majority of Hla. In total, these data suggest that MEDI4893 should have broad activity against Hla from MSSA and MRSA respiratory strains from diverse locations around the world.

## Supplementary Material

Supplemental material
