# Supplementary material for: Staphylococcus aureus Alpha-Toxin Is Conserved among Diverse Hospital Respiratory Isolates Collected from a Global Surveillance Study and Is Neutralized by Monoclonal Antibody MEDI4893
Source: Antimicrob Agents Chemother. 2016 Aug 22;60(9):5312–21. doi: 10.1128/AAC.00357-16 (PMC4997823; doi:10.1128/AAC.00357-16)
Supplement: Supplemental material [file supp_60_9_5312__index.html]

Staphylococcus aureus Alpha-Toxin Is Conserved among Diverse Hospital Respiratory Isolates Collected from a Global Surveillance Study and Is Neutralized by Monoclonal Antibody MEDI4893 — Supplemental material 

# Staphylococcus aureus Alpha-Toxin Is Conserved among Diverse Hospital Respiratory Isolates Collected from a Global Surveillance Study and Is Neutralized by Monoclonal Antibody MEDI4893

## Supplemental material

- Supplemental file 1 -

  Fig. S1

  PDF, 26K
