# Supplementary material for: Staphylococcus aureus Alpha-Toxin Is Conserved among Diverse Hospital Respiratory Isolates Collected from a Global Surveillance Study and Is Neutralized by Monoclonal Antibody MEDI4893
Source: Antimicrob Agents Chemother. 2016 Aug 22;60(9):5312–21. doi: 10.1128/AAC.00357-16 (PMC4997823; doi:10.1128/AAC.00357-16)
Supplement: Supplemental material [file AAC.00357-16_zac009165467so1.pdf]

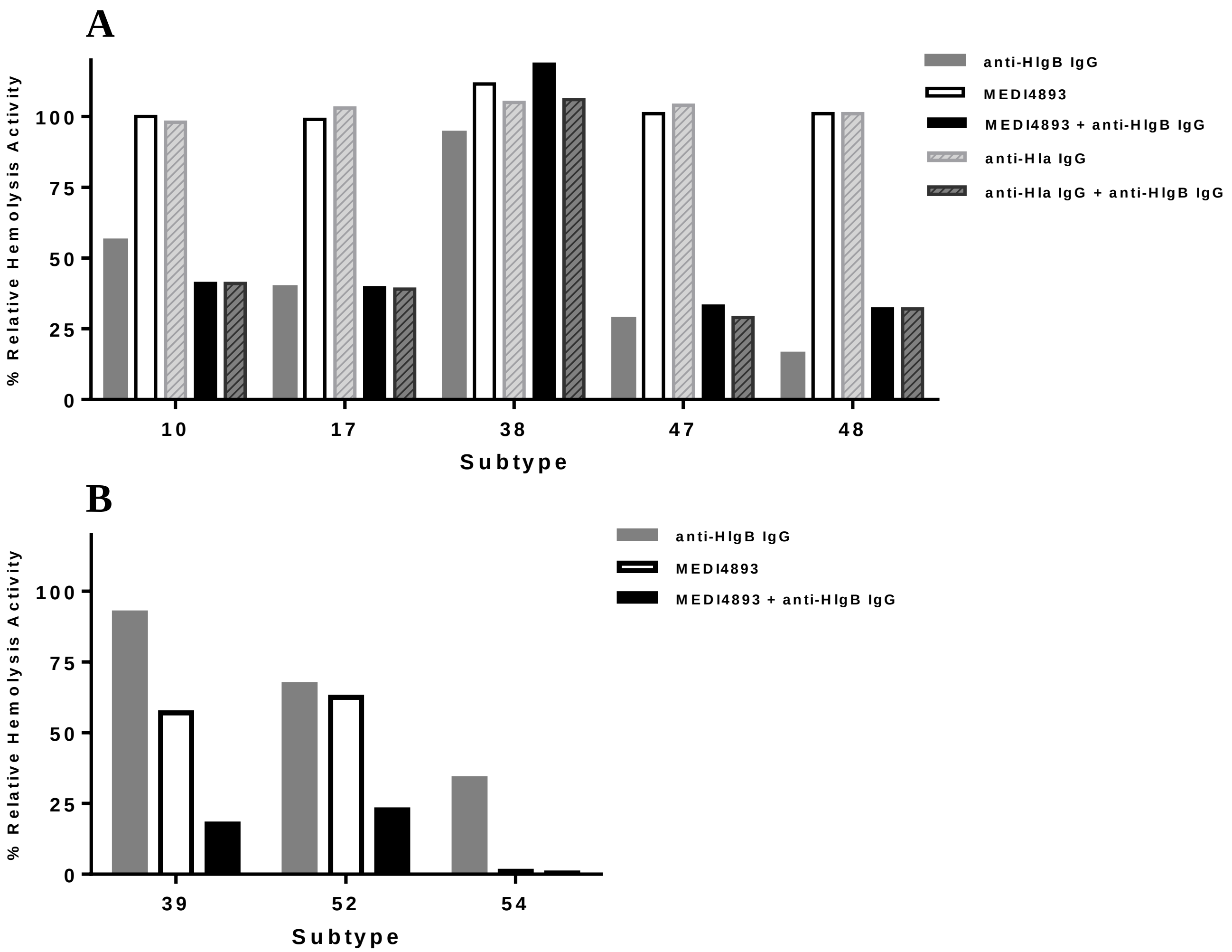

Supplementary Figure 1. Hemolytic activity of MEDI4893-resistant *S. aureus* supernatants is dependent on γ-hemolysin. Supernatants from selected *S. aureus* isolates that were not completely neutralized by MEDI4893 were incubated with either anti-HlgB purified rabbit IgG, anti-Hla purified rabbit IgG, or MEDI4893 alone or in combination compared to anti-chicken ovalbumin (OVA) purified rabbit IgG as a negative control. Percent relative hemolytic activity is shown for (A) HlgB lytic strains and (B) combined HlgB and Hla lytic strains.
